# Clinically Important Features of Porphyrin and Heme Metabolism and the Porphyrias

**DOI:** 10.3390/metabo4040977

**Published:** 2014-11-03

**Authors:** Siddesh Besur, Weihong Hou, Paul Schmeltzer, Herbert L. Bonkovsky

**Affiliations:** 1 Department of Medicine and Center for Liver Disease, Carolinas HealthCare System, Charlotte, NC 28204, USA; E-Mail: paul.schmeltzer@carolinas.org; 2 Department of Research and the Liver, Digestive, and Metabolic Disorders Laboratory, Carolinas HealthCare System, Charlotte, NC 28203, USA; E-Mail: weihong.hou@carolinas.org; 3 Department of Medicine, Universities of CT, Farmington, CT 06030 and North Carolina, Chapel Hill, NC 27599, USA; E-Mail: hbonkovsky@me.com

**Keywords:** 5-aminolevulinic acid, heme, iron, metalloporphyrins, mitochondria, porphobilinogen, porphyrias, porphyrins

## Abstract

Heme, like chlorophyll, is a primordial molecule and is one of the fundamental pigments of life. Disorders of normal heme synthesis may cause human diseases, including certain anemias (X-linked sideroblastic anemias) and porphyrias. Porphyrias are classified as hepatic and erythropoietic porphyrias based on the organ system in which heme precursors (5-aminolevulinic acid (ALA), porphobilinogen and porphyrins) are chiefly overproduced. The hepatic porphyrias are further subdivided into acute porphyrias and chronic hepatic porphyrias. The acute porphyrias include acute intermittent, hereditary copro-, variegate and ALA dehydratase deficiency porphyria. Chronic hepatic porphyrias include porphyria cutanea tarda and hepatoerythropoietic porphyria. The erythropoietic porphyrias include congenital erythropoietic porphyria (Gűnther’s disease) and erythropoietic protoporphyria. In this review, we summarize the key features of normal heme synthesis and its differing regulation in liver *versus* bone marrow. In both organs, principal regulation is exerted at the level of the first and rate-controlling enzyme, but by different molecules (heme in the liver and iron in the bone marrow). We also describe salient clinical, laboratory and genetic features of the eight types of porphyria.

## 1. Introduction

In this review, we first present an overview of normal heme synthesis and breakdown, with emphasis on the key roles played by the first and rate-controlling steps of heme synthesis and breakdown, catalyzed, respectively, by 5-aminolevulinic acid (ALA) synthase and heme oxygenase (HMOX). Next, we provide an update that includes descriptions of all eight of the human porphyrias, and we provide recommendations regarding their diagnosis and management. It is important that a clear diagnosis be made or that a diagnosis of porphyria is excluded early on when patients present with symptoms or signs suggestive of it. It is all too common to see patients, especially those with mild and non-specific symptoms and non-diagnostic mild to moderate increases in urinary porphyrins (usually mainly coproporphyrins) who have been erroneously labeled as having porphyria and who, too often, have been treated with intravenous heme administered by way of central venous catheters or ports. We hope that this review will reduce the number of false diagnoses in the future.

## 2. Key Features of Heme Metabolism and Regulation

### 2.1. Heme Biosynthesis and Its Regulation

#### 2.1.1. Heme Biosynthesis

**Figure 1:**
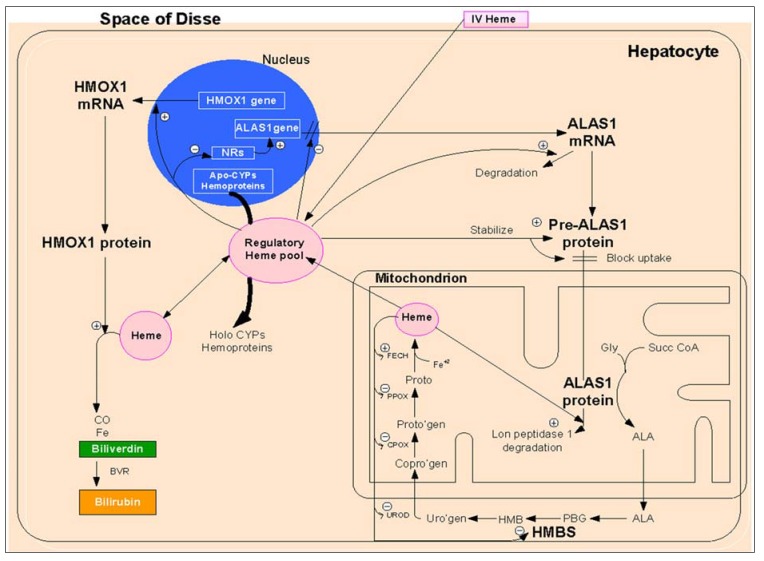
The heme biosynthetic pathway and aspects of its regulation in hepatocytes. Key roles are played by 5-aminolevulinic acid synthase-1 (ALAS1), heme oxygenase 1 (HMOX1), nuclear receptors (NRs) and hydroxymethylbilane synthase (HMBS) (also known as porphobilinogen (PBG) deaminase). Heme itself downregulates several steps in the synthetic pathway, especially ALAS1, by downregulating transcription, upregulating mRNA breakdown, blocking uptake into mitochondria and increasing Lon peptidase 1 breakdown of the mature mitochondrial enzyme. Heme upregulates HMOX1, mainly by increasing its transcription through binding to Bach1, a tonic repressor. HMBS is present in low amounts and becomes rate controlling when ALAS1 is induced. Fifty percent deficiency of HMBS, the defect in acute intermittent porphyria (AIP), can lead to critical deficiency of heme and uncontrolled induction of ALAS1. Heme administered intravenously is taken up well by hepatocytes and can replete heme pools rapidly and correct the defects caused by HMBS and other synthetic enzyme deficiencies. More recent studies in cell culture models suggest that heme excess may exert additional effects on other enzymes in the synthetic pathway, as suggested by the + and − symbols in the figure (World J Gastroent 19 (10): 1593). However, whether such effects are clinically relevant remain uncertain.

In humans and other higher animals, heme biosynthesis takes place mainly in hepatocytes and developing erythroid cells of bone marrow, and it is initiated by the formation of 5-aminolevulinic acid (ALA) from glycine (Gly) and succinyl-CoA, catalyzed by ALA synthase (ALAS), which is located in the matrix of mitochondria (Figure 1).

ALA exits the mitochondria through mitochondrial membranes, probably transported by still unidentified transport channels, and enters the cytosol, where two ALA molecules are joined together to form porphobilinogen (PBG) by the enzyme, ALA dehydratase (ALAD, also known as PBG synthase). Next, four molecules of PBG are polymerized by PBG deaminase (PBGD, also known as HMB synthase) to generate the linear tetrapyrrole hydroxymethylbilane (HMB). HMB cyclizes readily to form uroporphyrinogen (Uro’gen) I. However, under normal physiological conditions, little of the Uro’gen I isomer forms, because of the activity of the enzyme, Uro’gen III synthase (URO3S, also known as Uro’gen co-synthase). The Uro’gen undergoes decarboxylation by the cytoplasmic enzyme, uroporphyrinogen decarboxylase (UROD), to form coproporphyrinogen (Copro’gen). The Copro’gen formed is acted on by the enzyme, coproporphyrinogen oxidase (CPOX), to convert it into protoporphyrinogen. The next series of sequential decarboxylation reactions are carried out by a single cytosolic enzyme, called protoporphyrinogen oxidase (PPOX), to form protoporphyrin ;IX (PP). The final step of heme biosynthesis, catalyzed by the enzyme, ferrochelatase (FECH, also known as heme synthase), is the insertion of ferrous iron into PP to produce heme (Figure 1).

Under conditions of iron deficiency, lead poisoning or excessive formation of PP, zinc protoporphyrin (ZnPP) is formed as a by-product in the heme biosynthesis pathway, resulting from insertion of Zn^2+^ rather than Fe^2+^ into protoporphyrin by FECH. FECH is also capable of inserting cobalt into PP with the formation of cobalt protoporphyrin (CoPP), a metalloporphyrin with physiologic effects that are of interest. It is a potent suppressant of appetite and food intake. It is a long-acting inducer of HMOX 1 [1,2].

##### 2.1.1.1. Regulation of Heme Biosynthesis by ALA Synthase

The regulation of heme biosynthesis mainly occurs at its first step catalyzed by ALAS, the rate-limiting enzyme in the heme biosynthesis pathway (Figure 1, Table 1). There are two isoforms of ALAS, a ubiquitous housekeeping form (ALAS1), found in virtually all cells and especially in hepatocytes, and an erythroid form (ALAS2), which is the principal form in developing erythrocytes. Regulation of ALAS1 and ALAS2 enzymes in the heme biosynthesis varies between developing erythroid cells and hepatocytes.

**Table 1:** Rate-controlling enzymes of heme biosynthesis, catabolism and major mechanisms for their regulation.

Rate-Controlling Enzyme	Tissue Origin Subcellular Location	Gene and Chromosome Location	Gene Regulation
Heme Biosynthesis ALAS1 ALAS2	Ubiquitous Mitochondria Bone marrow Mitochondria	ALAS1 3p31.2 ALAS2 Xp11.2	Transcriptional regulation: Down-regulation by heme, glucose and sugars Induction by chemical, drugs, stress, circadian rhythm Post-transcriptional regulation: Destabilization of ALAS1 mRNA by heme Post-translational regulation: Impediment to pre-ALAS1 import into mitochondria Degradation of ALAS1 protein by heme Up-regulation by hypoxia and iron
Heme Catabolism HMOX1 HMOX2	Ubiquitous Mainly in smooth endoplasmic reticulum, mitochondria and nucleus Brain and testes Smooth endoplasmic reticulum	HMOX1 22q12.3	Transcriptional regulation: Up-regulation by heme and other metalloporphyrins Down-regulation by metalloporphyrins Induction by chemical and physical stresses Genetic polymorphisms Translational regulation: miRNAs Alternative splicing in the 5′-UTR

##### 2.1.1.2. Transcriptional Regulation

In non-erythrocytes, particularly in hepatocytes, heme-mediated feedback inhibition is the key feature of the regulation of ALAS1 at multiple levels. Transcription of ALAS1 is down-regulated by heme; for example, we first reported that heme repression of ALAS1 transcription is mediated by a region located between −6267 to −3447 upstream of the transcription starting point of the avian ALAS1 gene [3,4]. Glucose and other metabolizable sugars also down-regulate the ALAS1 gene expression at the transcriptional level, mainly acting through peroxisome proliferator-activated co-activator 1 α (PGC-1α), a co-activator of nuclear receptors and transcription factors [5,6,7,8]. Under fasting conditions, cellular glucose levels are low, the PGC-1α gene expression is up-regulated and, in turn, increases transcription of the ALAS-1 gene [9,10], which is likely the reason for fasting as a trigger of acute porphyria attacks and for the benefits of glucose infusions to attenuate the severity of acute attacks of porphyria [11,12]. In addition, PGC-1a is also a target of a circadian oscillator, the heme-sensing nuclear receptor Rev-erb alpha [13], and therefore, it may also contribute to the circadian regulation of ALAS1. In contrast toheme and glucose, a number of drugs and chemicals directly and markedly stimulate transcription of the ALAS1 gene through interactions of the nuclear receptors pregnane X receptor (PXR), constitutive androstane receptor (CAR) and chicken xenobiotic receptor (CXR) with drug-responsive elements located in the 5′ regulatory region of ALAS1 [14,15,16]. Indeed, certain drugs are among the well-documented precipitating agents of acute porphyric attacks.

##### 2.1.1.3. Post-Transcriptional Regulation

In humans, alternative splicing of ALAS1 in hepatic cells generates two forms of ALAS1 mRNA with distinct 5′-untranslated regions (5′-UTRs) [17,18]. Exon 1B is missing in a major form of the ALAS1 mRNA, while this exon is retained in a minor form of the ALAS1 mRNA [15]. Heme regulates the ALAS1 mRNA stability of the major form at the post-transcriptional level through enhancement of the breakdown of its mRNA, whereas the minor form is resistant to heme-mediated decay and may require translation for destabilization in response to heme [17,18].

##### 2.1.1.4. Post-Translational Regulation

Heme can also affect ALAS1 post-translationally. There is a putative heme-binding motif in the N-terminal mitochondrial targeting sequence of the pre-ALAS1 protein [19], and binding of heme to the motif results in decreased transport of pre-ALAS1 into mitochondria [20,21], where the translocation signal of sequence on the pre-ALAS1 protein is cleaved and processed into the mature form. Although both ALAS1 and ALAS2 contain the heme-binding motif, it is functional only in ALAS1 [21]. In addition, we have recently shown that heme increases the degradation of the mature, mitochondrial form of ALAS1 protein through proteolysis mediated by LONP1, an ATP-dependent protease that controls the selective turnover of mitochondrial matrix proteins [22].

### 2.2. Heme Catabolism and Its Regulation

#### 2.2.1. Heme Catabolism

The organs mainly involved in heme degradation are the spleen and liver. The initial step in heme breakdown opens the heme ring at the α-methene bridge and produces equimolar amounts of biliverdin, iron and carbon monoxide (CO) (Figure 1). Biliverdin is further converted to bilirubin by biliverdin reductase (BVR) and excreted from the liver to biliary canaliculi in most mammals.

Heme breakdown is a strictly controlled process, regulated by heme oxygenase (HMOX), the first and rate-controlling enzyme in the pathway of heme degradation (Figure 1). HMOX has two main isoforms: HMOX1 and HMOX2. The HMOX1 gene is expressed at relatively low levels, but is highly inducible by chemical and physical stress (e.g., reactive oxygen species (ROS), arsenicals, transition metals, heat shock), by heme, its physiologic substrate, and other selected metalloporphyrins (MePns) (Figure 1, Table 1). Unlike HMOX1, the HMOX2 gene is virtually non-inducible. It is highly and constitutively expressed in brain and testes, where its function may be principally to form carbon monoxide (CO), an important vasodilator and signaling molecule.

#### 2.2.2. Regulation of HMOX1

##### 2.2.2.1. Transcriptional Regulation

Regulation of the HMOX1 gene expression and the mechanisms underlying induction of HMOX1 by chemical and physical stresses are complex and regulated tightly at the transcriptional level by a number of transcription factors, including, but not limited to Bach1 [23,24,25], nuclear factor (erythroid-derived 2)-like 2, also known as NFE2L2 or Nrf2 [26], activating protein-1 (AP-1) [27,28], and others. These transcriptional factors interact with a number of regulatory elements located in in the 5′ up-steam promoter region of the HMOX1 gene, such as a series of expanded AP-1 sites, also called antioxidant responsive elements (AREs), Maf protein responsive elements (MAREs) and metalloporphyrin-responsive elements (MPREs), and regulate the HMOX1 gene expression. Bach1 is a basic leucine zipper (bZip) mammalian transcriptional repressor that negatively regulates the HMOX1 gene [23,24,25]. Bach1 is a sensor of heme and a heme-regulated transcriptional repressor. Under normal conditions, Bach1 forms antagonizing heterodimers with the Maf-related oncogene family. These heterodimers bind to Maf recognition elements (MAREs) in the HMOX1 promoter and suppress the HMOX1 gene expression. Under condition of excess intracellular heme, heme binds to the cysteine-proline (CP) motifs of Bach1 and leads to the dissociation of Bach1 from a heterodimeric repressor complex with small Maf proteins and relief of the repression, while the HMOX1 gene expression is upregulated [23,24].

We and others have recently found that a number of microRNAs (miRNAs), miRNA-196, miR-155 and *let-7* miRNA family members, interact with the binding sites in the 3’-UTR of Bach1 mRNA, which results in down-regulation of Bach1 protein expression and up-regulation of the HMOX1 gene in human hepatocytes [29,30,31,32]. Nrf2 is a basic leucine transcription factor and one of the major stimulatory Maf proteins, which positively regulates the HMOX1 gene expression [26]. Nrf2 forms heterodimers with Maf proteins. These heterodimers bind to antioxidant responsive elements (AREs) and induce HMOX1 gene expression [26].

Bach1 and Nrf2 compete for binding to AREs and exert their Bach1-dependent repressive or Nrf2-dependent activating effects on the ARE-mediated expression of genes encoding several antioxidant proteins, including HMOX1. The Bach1 repressive effect appears to dominate over the Nrf2 inductive effect, such that a tonic state of HMOX1 gene repression exists unless levels of nuclear Bach1 are decreased.

Genetic polymorphisms, including the length of guanosine-thymidine (GT) repeats and a single nucleotide polymorphism at position −413 (A/T, rs2071746) in the 5′-flanking region of the HMOX1 gene also influence its transcriptional expression [33,34,35,36]. It has been well established that longer (GT)_n_ repeats (>25 bases) in the 5′-flanking region of the HMOX1 gene are associated with lower HMOX1 gene expression, whereas shorter lengths of (GT)n repeats are associated with higher activities of HMOX1. The length of (GT)n repeats in this region has been directly correlated with susceptibility and outcomes of a variety of diseases, such as chronic obstructive pulmonary disease (COPD), coronary artery disease, diabetes mellitus and arthritis [32,33,35,36,37]. However, we and others found that the length of the GT repeats did not influence the course or severity of chronic hepatitis C [38].

##### 2.2.2.2. Translational Regulation

miRNAs, approximately 22 nucleotides (nt) in length, are a class of small non-coding RNAs that regulate gene expression, primarily through post-transcriptional repression [39,40,41]. Because of recent findings, it has become even more evident that miRNAs play a role in regulating HMOX1 gene expression, either directly or indirectly.

miR-217 and miR-377 in combination were found to attenuate HMOX1 protein expression, whereas knockdown of miR-217 and miR-377 up-regulates HMOX1 protein, but exhibits no alteration in HMOX1 mRNA levels, suggesting that the regulation occurs at the translational level [42]. In addition to translational regulation of HMOX1 by miRNAs, one study has recently suggested that a genetic factor, alternative splicing within the HMOX1 5′-UTR, is involved in regulation of human HMOX1 at the translational level and affects disease outcome [43].

## 3. The Porphyrias: Disorders of Heme Synthesis

The porphyrias are generally classified either according to the major site of overproduction of heme precursors (either liver or bone marrow) or according to the cardinal clinical features (either neuro-visceral or cutaneous) (Table 2). However, it is important to note that there is no one single simple classification of porphyrias, and clinical features of different forms may be similar. Hepatic porphyrias are disorders that result from overproduction of porphyrins or porphyrin precursors chiefly in the liver due to enzymatic defects in heme synthesis. The hepatic porphyrias are further classified as acute or inducible porphyrias and chronic hepatic porphyrias. This is based on the acuity of clinical manifestations and does not signify the duration of the disease. The acute hepatic porphyrias include acute intermittent porphyria (AIP), hereditary coproporphyria (HCP), variegate porphyria (VP) and porphyria due to severe deficiency of 5-aminolevulinic acid (ALA) dehydratase (ALADP) [44,45]. The chronic hepatic porphyrias are porphyria cutanea tarda (PCT) and hepatoerythropoietic porphyria (HEP).

### 3.1. Acute Porphyrias: Acute Intermittent Porphyria as a Paradigm

#### 3.1.1. Epidemiology

In most countries, AIP is the most common and 5-aminolevulinic acid (ALA) dehydratase (ALAD) porphyria the least common acute porphyria. All acute porphyrias are autosomal dominant with the exception of ALADP, which is an autosomal recessive disorder (Table 2) [46,47]. Acute intermittent porphyria affects people of all races and regions, but it is more common in people of Northern European descent. The incidence is four-fold higher in Sweden than in rest of Europe, because of a founder effect [48]. The overall prevalence of clinically manifest AIP is estimated to be ~50–500/million; however, only 10–20/million [49,50,51,52,53] have clinically overt AIP with penetrance of ~4%–20%. The penetrance seems to be much lower in non-familial AIP. This was highlighted by Nordmann *et al.*, who evaluated the prevalence of AIP genotype in French blood donors. 

They found that clinical penetrance of AIP mutations was ten times lower than suggested by family studies [54]. A recent study by Elder *et al.* [55] evaluated the incidence and prevalence of porphyrias in Europe using the information collected prospectively by the European Porphyria Network. The incidence of symptomatic AIP was similar in all countries (0.13 per million per year; 95% CI: 0.10–0.14), except Sweden (0.51; 95% CI: 0.28–0.86). Only 3%–5% of patients with AIP were estimated to develop acute attacks. The incidence rate for VP was about half that of AIP and four times greater than HCP. Most patients with VP (80%) presented with dermatologic symptoms. An unexplained higher prevalence was found in Switzerland [56], inconsistent with prior estimates. The incidence and prevalence of the cutaneous porphyria, erythropoietic protoporphyria (EPP), varied between countries, with the incidence ranging from 0.03 to 0.36 per year per million. This disparity may be due to increased clinical awareness, wider availability of better diagnostic methods and partly due to variation in pigmentation.

**Table 2:** Classification of the Porphyrias.

**A. According to the Clinical Manifestations of Disease**					
	**Type**	**Inheritance**	**Gene Affected**	**Chromosomal Location**	**Comments**
**Acute or Inducible Porphyrias**	ALADP	AR	*Pbgs*	9q34	Very rare severe disease in infancy
	AIP	AD	*Hmbs*	11q23.3	Most severe form
	HCP	AD	*Cpox*	3q12	May also have cutaneous features
	VP	AD	*Ppox*	1q22	May also have cutaneous features
**Cutaneous Chronic Porphyrias**	CEP	AR	*Uro3*	10q26.1-q26.2	Rare usually manifests itself in infancy/childhood
	HEP	AR	*Urod*	1p34.1	Rare usually manifests itself in infancy/childhood
	PCT (Type I)	Acquired	None		Diseases of adults
	PCT (Type II)	AD	*Urod*	1p34.1	Requires additional defects
	EPP	AR	*Fech*	18q21.31	Common; onset in infancy
	XLPP	X-linked	*Alas1*	Xp11.21	Gain of function mutations
**B. According to the Clinical Manifestations of Disease**					
****	**Type**	**Inheritance**	**Gene Affected**	**Chromosomal Location**	**Comments**
**Acute Hepatic Porphyrias**	ALADP	AR	*Pbgs*	9q34	Very rare severe disease in infancy
	AIP	AD	*Hmbs*	11q23.3	Most severe form
	HCP	AD	*Cpox*	3q12	May also have cutaneous features
	VP	AD	*Ppox*	1q22	May also have cutaneous features
**Chronic Hepatic Porphyrias**	PCT (Type I)	Acquired	None		Diseases of adults
	PCT (Type II)	AD	*Urod*	1p34.1	Requires additional defects
	HEP	AR	*Urod*	1p34.1	Rare usually manifests itself in infancy/childhood
**Erythropoietic Porphyrias**	CEP	AR	*Uro3*	10q26.1-q26.2	Rare usually manifests itself in infancy/childhood
	EPP	AR	*Fech*	18q21.31	Common; onset in infancy

AD, autosomal dominant; CEP, congenital erythropoietic porphyria; ADP, ALA dehydratase deficiency porphyria; EPP, erythropoietic protoporphyria; AIP, acute intermittent porphyria; HEP, hepatoerythropoietic porphyria; AR, autosomal recessive; PCT, porphyria cutanea tarda; VP, variegate porphyria.

The disease manifests typically in adult women in their second through fourth decades of life even though, in all likelihood, both genders equally inherit PBGD mutations. Manifestation of AIP is very uncommon before puberty. In one prospective study using the Swedish registry, children (3–16 years) with established mutations for AIP were observed for 2.5 years. Only 10% developed vague symptoms like abdominal pain, nausea, *etc.* The symptoms were mild and lasted only for a short duration. It is unclear whether these symptoms arose due to porphyrias or to another cause [57].

#### 3.1.2. Pathogenesis of Acute Attacks

Patients with AIP, HCP and VP have partial (50% of the normal) enzymatic deficiency as opposed to porphyria due to severe deficiency of ALA dehydratase (<5% of the normal). Affected individuals with these enzymatic defects become susceptible to acute attacks of porphyria. 

Acute porphyric attacks can be triggered by severe fasting or dieting, alcohol, certain drugs (especially barbiturates, hydantoins, rifampin, sulfonamides and endogenous steroid hormones, estrogen and progesterone) and other intercurrent illnesses or stress (Table 3).

**Table 3:** Factors known to trigger or exacerbate acute attacks of porphyria [11].

Exacerbating Factors	Common Unsafe Drugs
Drugs and chemicals—especially	➢ Excess alcohol
	➢ Barbiturates
	➢ Estrogens
	➢ Hydantoins
	➢ Progestagens
	➢ Sulfonamides
	➢ All drugs that are suicide substrates or potent inducers of cytochrome P450
Dieting; fasting; deficiency of carbohydrate intake (gastric bypass surgery)	-
Exhaustion—emotional or physical	-
Intercurrent acute illnesses	-

Note: More extensive list of drugs and their status are available from the websites of American Pophyria Foundation [58], European Porphyria Network [59] and University of Cape Town Poprhyria Service [60]

More extensive lists of drugs that may trigger acute attacks are available at the following websites: the American Porphyria Foundation website [58] the European Porphyria Network website [59]) and the University of Cape Town, South Africa, website [60]).

The biochemical hallmark of acute porphyric attacks is marked up-regulation of hepatic ALA synthase-1 (Figure 1). This may occur due to drugs and chemicals, such as barbiturates, hydantoins and alcohol, which directly up-regulate expression of the gene and which also increases heme demand by up-regulating hepatic cytochrome P-450 (Figure 1). Fasting/starvation also up-regulates hepatic ALA synthase-1, as already described. The exact mechanism for the neurologic damage in acute porphyrias is not completely understood. Several hypotheses, including elevated levels of ALA, defects in heme synthesis in neural tissue and elevated levels of tryptophan and serotonin have been proposed as possible causes for the neurovisceral features of acute porphyric attacks. 

However, the preponderance of evidence favors ALA (or perhaps, a metabolite of ALA) as the chief neurotoxin [61,62]. Recent observations of biochemically active AIP occurring in men who received livers removed from women with severe, symptomatic AIP is further evidence for the importance of the liver as the key organ in pathogenesis.

Acute attacks commonly occur in women between the second and fourth decades of life. The attacks are often linked to menstrual cycles, highlighting the importance of endogenous steroids, especially progesterone, in pathogenesis. However, pregnant women typically do not suffer severe attacks, despite increased levels of estrogen and progesterone [63]. Rather, acute attacks tend to occur in the post-partum period, for unknown reasons.

#### 3.1.3. Clinical Features

The clinical features are similar during acute porphyric attack in all acute porphyrias. However, the attacks are less frequent and less severe in variegate and hereditary coproporphyria [64]. Most clinical manifestations are due to the effects of precursors of heme on the nervous system. Colicky abdominal pain is the most common presenting symptom, usually affecting the lower abdomen and lasting hours to days. It is gradual in onset and escalates in severity. Other frequent symptoms, signs and their characteristics are listed in Table 4 [64,65,66]. Common neurological manifestations of acute porphyrias include severe pain, paresis and peripheral neuropathy. Affected individuals may also present with muscle weakness, difficulty swallowing, other bulbar signs, confusion, delirium and seizures. Generalized weakness may sometimes progress rapidly to quadriparesis and acute respiratory insufficiency, especially if the correct diagnosis is missed and patients are treated with barbiturates or hydantoins (e.g., for treatment of seizures). Neurologic manifestations of acute porphyrias are listed in descending order of frequency in Table 4. Many patients report passing red to brown urine that may darken when exposed to air, light and warmth. Such findings should alert clinicians to consider the diagnosis of acute porphyria. Hyponatremia is a common electrolyte abnormality that occurs during acute attacks. Factors that contribute to hyponatremia include syndrome of inappropriate antidiuretic hormone secretion (SIADH), vomiting and resuscitation with high volumes of dextrose solutions given intravenously [67]. Skin manifestations do not occur in acute intermittent porphyria with the rare exception of patients with end stage renal disease, in whom levels of porphyrins increase in the plasma. They may develop blistering skin lesions [68]. Cutaneous manifestations may occur in active HCP or VP due to accumulation of coproporphyrins or harderoporphyrin (a porphyrin with three carboxyl groups, intermediate between copro-and proto-porphyrin).

### 3.2. Diagnosis

The clinical presentations of acute porphyrias are nonspecific in the absence of pathognomonic signs or symptoms. Hence, laboratory evaluation is vital for diagnosis of acute porphyria. Qualitative or semi-quantitative urinary porphobilinogen (PBG), performed on a single random urine sample, is themost important rapid test for diagnosis of acute porphyria [69]. The results from tests, such as the Watson–Schwartz test or the Hoesch test, in theory, can be available rapidly as the tests take less than 10 min to perform. Unfortunately, in the era of CLIA-certified (Clinical Laboratory Improvement Act) labs in the U.S., such tests currently are not available in most hospitals or urgent care clinics.

**Table 4:** Common presenting symptoms and signs of acute porphyric attacks.

Symptoms and Signs	Estimated Symptoms and Signs Incidence, (%)	Comment	
**Gastrointestinal**			
Abdominal Pain	85–95		Usually unremitting (for hours or longer) and poorly localized, but can be cramping.
Vomiting	43–88		Neurologic in origin and rarely accompanied by peritoneal signs, fever or leukocytosis. Nausea vomiting often accompanies abdominal pain.
Constipation	48–84		May be accompanied by bladder paresis.
**Neurologic**			
Pain in extremities and/or back	50–70		Pain may begin in the chest or back and move to the abdomen. Extremity pain, chest, neck or back indicates involvement of sensory nerves, with objective sensory loss reported in 10%–40% of cases.
Paresis	42–68		May occur early or late during a severe attack.
Respiratory Paralysis	9–20		Muscle weakness usually begins proximally rather than distally and more often in the upper than lower extremities, preceded by progressive peripheral motor neuropathy and paresis.
Mental Symptoms	40–58		May range from minor behavioral changes to agitation, confusion, hallucinations and depression
Convulsions	10–20		A central neurologic manifestation of porphyria or due to hyponatremia, which often results from syndrome of inappropriate antidiuretic hormone secretion or sodium depletion.
**Cardiovascular**			
Tachycardia	64–85		May warrant treatment to control rate, if symptomatic.
Systemic arterial hypertension	36–55		May require treatment during acute attacks and may sometimes become chronic.

In subjects with compatible symptoms and signs, a positive urinary PBG screening test establishes the diagnosis of acute porphyria. It should be confirmed by measuring quantitative ALA, PBG, total porphyrins and creatinine in the same urine sample that was used for the initial rapid screening test. In anuric patients, diagnosis of acute porphyria can be established by measuring serum PBG. All patients with true manifestations of AIP will have markedly increased serum and urinary ALA and PBG levels during an attack (up to 25–100 mg of ALA and 50–200 mg PBG in urine per day, normal 0–4 mg/day). An alternate diagnosis, such as ALADP, HCP, VP, lead poisoning or hereditary tyrosinemia type 1, should be considered if urinary ALA excretion (in mg/g creatinine) exceeds that of PBG. 

PBG in urine may be converted non-enzymatically to uroporphyrin, and hence, although the defect in AIP lies in hepatic PBG deaminase, there may be increased urinary uro- and copro-porphyrin levels. Urine may turn pink or even dark red or black (due to porphyrin or porphobilin formation) following exposure to air and light [70,71,72].

The major increases in porphyrins and their precursors that typically occur in biochemically manifest porphyrias are summarized in Table 3. The second line of diagnostic evaluation includes testing for plasma and urine fecal porphyrins, erythrocyte PBG deaminase levels, along with DNA genetic analysis for mutation in the relevant genes. AIP can be differentiated from other porphyrias by measuring erythrocyte PBG deaminase levels. However, this alone cannot be used for diagnosis of AIP, because various factors, like gene mutations causing selective PBG deaminase deficiencies in liver with normal levels in erythrocytes or an increase in erythrocyte enzyme levels due to concomitant hemolytic anemia with reticulocytosis, may lead to false negative results (PBGD levels are higher in immature RBCs). This occurs in ~5% of subjects with AIP. Molecular methods and DNA analysis for gene mutations to identify gene encoding PBG deaminase are not only helpful in confirming AIP, but also assist in identifying other gene carriers in the family. Targeted DNA analysis, which is less costly and time consuming, assists with the diagnosis when a specific mutation within a family or geographic area is already known. Lists of updated mutations are available at the human gene mutation database [73].

Laboratory data must be reviewed and confirmed irrespective of the degree of clinical suspicion. Nonspecific mild elevations in urine porphyrins occur in a number of conditions, including patients with hepato-biliary disease and lead toxicity. Fecal porphyrins may be increased in those with gastrointestinal bleeding or in patients who consume large amounts of red meat. Increased erythrocyte porphyrins, especially zinc protoporphyrin, may be seen in patients with iron deficiency and/or lead poisoning. Poor lab techniques, erroneous interpretation or lack of clinical experience commonly results in misdiagnosis. A recommended approach to make a correct diagnosis of acute porphyria and to distinguish among the several types accurately is outlined in Table 5.

#### Management

All acute hepatic porphyric attacks are managed in a similar fashion. Treatment should be initiated immediately in patients with well**-**documented acute porphyria who present to the emergency department with typical clinical features and without high fever, white cell count or peritoneal signs. They should be treated immediately with a high carbohydrate intake (at least 300 g/day), narcotic analgesics and phenothiazines. Patients with nausea and/or vomiting will need to receive dextrose with sodium and potassium intravenously, and fluid requirement will vary from patient to patient. Many patients with acute attacks can have electrolyte abnormalities, including hypomagnesemia and hyponatremia. Thus, it is vital to frequently monitor electrolytes. Changes in the rate and type of intravenous fluid administered should be made based on electrolyte values.

Initial management of acute porphyria also includes a search for and avoidance of precipitants and discontinuation of all potentially harmful drugs.

Many of the commonly used drugs can worsen symptoms or even have deleterious effects. Therefore, it is advised that providers consult the websites of the American Porphyria Foundation [58] the European Porphyria Network [59] and/or the South African [60] and Scandinavian Porphyria websites for information about the risks or safety of drugs in acute porphyrias.

Beta blockers, if not contraindicated, are used to treat tachycardia or hypertension secondary to sympathetic over-activity. Chlorpromazine, promethazine or ondansetron are the preferred antiemetics. Narcotic analgesics are used in the treatment of pain. Seizures are treated by gradual correction of hyponatremia, hypomagnesemia and safe anticonvulsants (gabapentin, vigabatrin and levetiracetam). The other measures include providing at least 300 g carbohydrate per day (enterally or parenterally), hydration and correction of electrolyte abnormalities. Patients with acute porphyric attacks should be closely monitored and if they show signs or symptoms of progressive confusion, delirium or weakness, they should be admitted to the intensive care unit.

**Table 5:** Enzymatic defects and major biochemical abnormalities in porphyrias. Only major increases in urine, stool, plasma and erythrocytes (RBCs) are shown.

Type of Porphyria	Enzyme Defect	Urine	Stool	Plasma	RBCs
X-linked protoporphyria	ALA synthase-2 (gain of function)	Normal	PROTO	PROTO	Zn PROTO
ALA dehydratase deficiency (ADP)	ALA dehydratase	COPRO ALA	Normal	ALA	Zn PROTO
Acute intermittent porphyria (AIP)	PBG deaminase	ALA, PBG, URO I	Normal COPRO I	ALA, PBG, URO I	↓PBGD
Congenital erythropoietic porphyria (CEP)	Uroporphyrinogen III synthase (cosynthase)	COPRO I URO I	COPRO I	COPRO I URO I	COPRO I URO I
Porphyria cutanea tarda (PCT) and Hepatoerythropoietic porphyria (HEP)	Uroporphyrinogen III decarboxylase	Uroporphyrin, heptacarboxyl porphyrin	Heptacarboxyl porphyrin ISOCOPRO	Uroporphyrin, heptacarboxyl porphyrin	Zn PROTO
Hereditary coproporphyria (HCP)	Coproporphyrinogen III oxidase	ALA, PBG, COPRO III	COPRO III	COPRO	Normal
Variegate porphyria (VP)	Protoporphyrinogen oxidase	ALA, PBG, COPRO III	PROTO COPRO III	Porphyrin peptide conjugate	Normal
Erythropoietic protoporphyria (EPP)	Ferrochelatase	COPRO III with hepatopathy	PROTO	PROTO	PROTO

Abbreviations: COPRO, coproporphyrin; URO, uroporphyrin; PROTO, protoporphyrin; Zn, zinc.

In moderate to severe attacks of acute porphyria, intravenous heme is the treatment of choice. Heme for human use is available in the U.S. as lyophilized hydroxy heme (Panhematin, Recordati Rare Chemicals) and in Europe, South Africa and some other countries as heme arginate (Normosang, Orphan Europe). In the U.S., Panhematin can be obtained within 12–24 h by calling 888-514-5204 (M–F, 9 am–6 pm ET) or 800-673-6723 (at all other times).

Administration of intravenous heme results in reduced activity of ALAS-1 (Figure 1). This, in turn, results in rapid reduction of hepatic overproduction of ALA and PBG. Typically, about 4–5 days of treatment with heme, 3 mg/kg BW/day, is required to resolve symptoms following an acute attack. Panhematin is supplied as a lyophilized powder cake in amber vials. It can be reconstituted either with sterile water or albumin. When reconstituted with sterile water, it should be administered quickly (within 1 h), because of the inherent instability of aqueous solutions. Most authorities recommend that it be reconstituted with albumin (132 mL of 25% human serum albumin) as per recommendations of United States Porphyria Consortium. Reconstitution with albumin improves stability (at least for 24 h) and reduces side effects [74,75].

Side effects of heme therapy include obliterative thrombophlebitis, coagulopathy, tachyphylaxis and iron overload when used frequently or for long periods (>3 years). Patients with frequent, nearly continuous and unremitting attacks should be considered for orthotopic liver transplantation. Those who undergo successful transplantation show rapid and complete, permanent resolution of symptoms and biochemical abnormalities [76,77,78]. In a few instances, the livers removed from women with severe AIP have been transplanted into older men with end-stage liver disease (domino liver transplant). Unfortunately, such men have developed biochemically and clinically active AIP, providing additional strong evidence that AIP is a metabolic liver disease.

### 3.3. Hereditary Coproporphyria (HCP)

#### 3.3.1. Pathogenesis

Hereditary coproporphyria is an autosomal dominant disorder that occurs as a result of deficiency of the mitochondrial enzyme coproporphyrinogen oxidase (CPOX).

#### 3.3.2. Clinical Features

Clinical features manifest when there is partial (~50%-heterozygous) or nearly complete (homozygous) deficiency of the CPOX enzyme. Neurovisceral manifestations are similar to, but less severe than, AIP. Cutaneous features are seen in ~30% of patients. These include vesiculo-bullous eruption, resembling that in PCT or HEP, usually involving the face, hands or other Sun-exposed skin areas. These lesions generally heal, often with scarring, changes in pigmentation or hypertrichosis.

#### 3.3.3. Diagnosis

Diagnosis of HCP should be considered if there is elevation of urinary ALA and PBG. However, unlike AIP, ALA excretion often exceeds that of PBG, and these levels usually normalize more quickly between attacks. Elevation of fecal coproporphyrin III alone without elevation of protoporphyrin IX differentiates it from VP. The fecal and urinary coproporphyrin III levels typically increase10–200-times the normal levels. Diagnosis can be confirmed by identifying the CPOX mutation.

#### 3.3.4. Management

Acute attacks of HCP are treated in a similar fashion to AIP, as already described. 

### 3.4. Variegate Porphyria (VP)

#### 3.4.1. Pathogenesis

VP is an autosomal disorder caused by a heterozygous deficiency of ~50% protoporphyrinogen oxidase (PPOX) activity with variable penetrance. The gene for this enzyme has been localized to chromosome 1q 22. It is predominantly a cutaneous disease (60%) with blistering lesions on Sun-exposed areas of the skin, but an appreciable proportion of patients (40%) also have neuro-visceral symptoms, as seen in acute porphyrias.

#### 3.4.2. Clinical Features

Most patients with the genetic defect in PPOX are asymptomatic most of the time. Bullae, erosions and ulcers may develop at an early age after minimal trauma to light-exposed skin. Acute neurovisceral attacks occur especially in women of child-bearing age and in those who have other risk factors, as already described.

#### 3.4.3. Diagnosis

Diagnosis of VP is made by a combination of blood, urine and stool tests. Patients with biochemically active VP will have increased PBG in a spot urine sample and/or increased plasma porphyrins, with distinctive fluorescence peak at approximately 626 to 628 nm at neutral pH. The latter is of much help in differential diagnosis, although it is not observed in all subjects. Stool studies reveal increased fecal porphyrins with a predominance of both coproporphyrin III and protoporphyrin IX. The diagnosis of VP should be confirmed by demonstrating a genetic mutation in PPOX or decreased PPOX activity in cells, such as lymphocytes.

#### 3.4.4. Management

Treatment of acute attacks of VP is similar to management of other acute hepatic porphyrias. Patients with cutaneous manifestations should limit Sun exposure, wear protective clothing and use sunscreens that block long-wave ultraviolet and blue light. Antibiotics should be used to treat skin infections, and topical steroids should be avoided.

### 3.5. 5-Aminolevulinic Acid Dehydratase Deficient Porphyria (ALADP)

#### 3.5.1. Pathogenesis

ALADP is a very rare autosomal recessive disorder resulting from severe deficiency of ALAD. The defect appears to be localized to the gene located on chromosome 9q34. Clinical features manifest if there is a profound (>90%) deficiency of ALAD. It is a highly heterogeneous disease, and 11 gene mutations [79] have been identified.

#### 3.5.2. Clinical Features

The complete range of clinical manifestations of ALAD is not well known, because of the paucity of cases reported so far. The presentation is very similar to other acute porphyrias. It includes neurovisceral symptoms, such as vomiting, abdominal pain, neuropathy and paresis. Sometimes, the paresis is chronic.

#### 3.5.3. Diagnosis

ALADP should be suspected when patients present with typical symptoms and urine tests show elevated urinary ALA and total porphyrins without an increase in PBG. Diagnosis is established by documenting ALAD deficiency in red blood cells and eliminating other potential causes (lead toxicity and tyrosinemia Type I).

#### 3.5.4. Management

Treatment of acute attacks is similar to management of other acute hepatic porphyrias. Withdrawal and avoidance of drugs and precipitating factors known to be harmful in other acute porphyrias is recommended.

### 3.6. Chronic Hepatic Porphyrias

These are porphyric conditions secondary to uroporphyrinogen decarboxylase (UROD) deficiency resulting in accumulation of highly carboxylated porphyrins, uro- and hepta-carboxyl porphyrins.

#### 3.6.1. Porphyria Cutanea Tarda (PCT)

##### 3.6.1.1. Epidemiology

PCT is the most common porphyria encountered in clinical practice. PCT has global prevalence and affects one in 20,000 in the United States [80]. It commonly affects men who are middle-aged (30–60 years) and very rarely affects children. Patients with *UROD* and *HFE* mutations may have early disease onset [81].

##### 3.6.1.2. Classification of PCT

PCT is classified into three types based on inheritance patterns and sites of UROD deficiency. All three forms have similar clinical features and are managed in a similar fashion. Moreover, in all forms, there are shared risk factors that trigger clinical manifestations.

Type I: This occurs due to acquired deficiency of UROD and usually is reversible. This is the most common type of PCT and comprises 70%–80% of cases [82]. Deficiency of UROD activity is noted in the liver, but non-hepatic tissues are unaffected.

Type II (familial): This results from the hereditary heterozygous mutation of one allele of the *UROD* gene. It affects 20% of patients with PCT. The affected patients have inherited 50% reduction of the UROD activity in the liver, red blood cells, fibroblasts and other tissues. 

This degree of reduction is insufficient to cause clinical disease. Other risk factors are also required for the disease to be clinically manifested (Table 4).

Type III: This affects <5% of patients with PCT. Such patients have a positive family history, normal erythrocyte UROD activity and decreased hepatic UROD activity. The nature of the underlying genetic defect remains unknown.

##### 3.6.1.3. Pathogenesis

Uroporphyrinogen decarboxylase (UROD) is found in the cytosol and catalyzes decarboxylation of uroporphyrinogen to coproporphyrinogen. PCT becomes clinically manifest following reduction of UROD to approximately 20% of normal. Based on murine models, it has been postulated that even in patients with heterozygous mutation (50% UROD activity), other factors and the generation of the UROD inhibitor, uroporphomethene [83], are necessary for PCT to develop. Risk factors (Table 6), like alcohol, estrogen, other drugs and chemicals, cause the induction of the enzyme, ALA synthase. The increased induction of ALA synthase I in the setting of UROD deficiency results in increased production of uroporphyrinogen I or III and may be catalyzed by CYP1A2 in the presence of iron [83], forming uroporphomethene that further inhibits UROD. However, iron does not directly inhibit hepatic UROD.

Hepcidin production is reduced by hepatitis C and mutations in *Hfe*, *Hjv* and *Tfr2* genes. The decrease in hepcidin leads to increased absorption of iron from the small intestine, leading to further iron overload and to amplification of metabolic disarray.

##### 3.6.1.4. Clinical Features

Patients with PCT predominantly develop cutaneous manifestations and do not develop any neurovisceral manifestations. The cutaneous lesions are gradual in onset, unlike those of EPP. The skin manifestations are mainly in the Sun-exposed areas (face, hands, forearms and lower legs). The skin lesions develop following minor trauma to the Sun-exposed areas and are not due to acute photosensitivity.

**Table 6:** Factors known to trigger or exacerbate PCT.

Exacerbating Factors	Common Drugs/Chemical Triggers
Alcohol excess with alcoholic liver disease	
Chronic hepatitis C	
Human immunodeficiency virus infection	
Mutations in the *Hfe*, *Hjv* and *Tfr2*	
End stage renal disease	
Drugs and chemicals, but especially	➢ Excess alcohol
	➢ Estrogens
	➢ Polyhalogenated aromatic chemicals

Patients can present with bullae, blisters, sores and vesicles. The bullae contain porphyrin-rich serous or serosanguinous fluid and rupture easily. 

The involved areas become crusted, can become secondarily infected and tend to heal with areas of hypo- or hyper-pigmentation or sclerodermatous change. Hirsutism and hyperpigmentation of skin in the Sun-exposed areas have been noted. Hepatic manifestations of PCT include elevations in serum aminotransferases and gamma glutamyl transpeptidase. Evidence of iron overload (elevated serum ferritin and transferrin saturation) is seen in most patients with PCT [68]. There is a strong association between hepatitis C and PCT [84,85]. However, there is a variation in prevalence based on geographic location. Patients within the United States (56%) and Southern Europe have a higher prevalence of hepatitis C as compared to those in Northern Europe and East Asia [86,87]. PCT patients with advanced fibrosis or cirrhosis are more often associated with hepatitis C as compared to patients with less advanced liver disease. HCV is thought to be a strong trigger for the development of deranged porphyrin metabolism in those with other known predisposing factors. As many as 15%–40% of patients with PCT develop cirrhosis [88], depending on the presence of other risk factors, like advanced age, heavy alcohol intake, hepatitis C, hemochromatosis and the degree of iron overload. Patients with PCT are also at high risk of developing hepatocellular carcinoma [89], probably higher than the risk of such development in patients with underlying chronic liver disease without PCT].

##### 3.6.1.5. Diagnosis

PCT should be considered in patients with blistering skin lesions in Sun-exposed areas. Several other porphyrias (VP, HCP) have similar skin lesions; hence, diagnosis should be confirmed by biochemical tests. Total serum and urine porphyrin levels should be checked. If the total porphyrins are elevated, additional testing should include plasma porphyrins, fractionated urine and or plasma porphyrins, urinary ALA, PBG, erythrocyte total porphyrins and fecal total porphyrins. In patients with PCT plasma and urine porphyrins, predominantly uro-, hepta- and hexacarboxyl-porphyrins, along with fecal isocoproporphyrins, are elevated.

##### 3.6.1.6. Management

Patients diagnosed with PCT should avoid exposure to direct sunlight, drugs and chemicals (Table 4) that can trigger or exacerbate PCT. Use of opaque sunscreen containing zinc or titanium oxide and use of protective clothing is recommended.

The following treatments are demonstrated to be helpful in the management of PCT: 

Iron reduction: In patients with established PCT and iron overload, therapeutic phlebotomy is recommended [90]. Such patients should undergo phlebotomy on a weekly basis until serum ferritin is <25 ng/mL. Failure to respond to phlebotomy suggests another type of porphyria. Phlebotomies may safely be discontinued after achieving remission and achievement of target levels of serum ferritin. However, in patients with *Hfe* genotypes C282Y/C282Y or C282Y/H63D, serum ferritin should be monitored, and they should undergo phlebotomies chronically to maintain serum ferritin below 100 ng/mL [91].

Oral or parenteral iron chelation therapy may be considered in those with underlying anemia or poor venous access. Iron chelation therapy should not be considered as initial treatment in the management of PCT, as it is not as effective as phlebotomies or cinchona alkaloids [92,93]. 

However, iron chelation may be considered in certain cohorts of patients with poor venous access, in those with underlying anemia or in patients with intolerance or contraindications to treatment with chloroquine or hydroxychloroquine. Orally available iron chelators include deferasirox (Exjade, Novartis) and deferiprone (Ferriprox, Apotex). The usual dose of deferasirox is 10 mg/kg BW/d. This therapy is much more expensive and has potential adverse effects (e.g., skin rash, nephrotoxicity, hepatotoxicity, especially as total body iron levels fall). Deferoxamine (Desferal, Novartis) is also effective, but less often considered, because it must be administered parenterally, usually nightly by subcutaneous infusion with a pump; it is expensive and it too has potential adverse effects (e.g., auditory (cranial nerve 8)) toxicity and development of cataracts. The iron chelators are all more expensive than therapeutic phlebotomies, and they have more potential adverse effects. Therefore, their use should generally be restricted to small and special cohorts (those with poor venous access, underlying anemia and those with contraindication or intolerance to antimalarials) [94].

Chloroquine or hydroxychloroquine: Low-dose hydroxychloroquine or chloroquine are commonly used antimalarials. One of these agents is used when phlebotomies are difficult or tolerated poorly [95]. Antimalarials mobilize the porphyrins from the liver by gaining access to the porphyrin-rich lysosomal compartment of hepatocytes and forming water-soluble complexes, which are excreted in the urine. Such treatment typically leads to remission of the disease after several months of treatment [95]. The effectiveness of hydroxychloroquine, 100 mg twice weekly, in terms of time to remission and safety profile was found to be similar to that of phlebotomy in a recent prospective study [93]. The exact duration of antimalarial therapy is unclear, but it should be continued at least until normalization of plasma and urine porphyrin levels. Antimalarials are contraindicated in pregnant or lactating women, subjects with glucose-6-phosphate dehydrogenase deficiency; psoriasis, retinal disease and should be used with special caution in those on potential hepatotoxic drugs (isoniazid, valproic acid).

### 3.7. The Erythropoietic Porphyrias

The erythropoietic porphyrias include congenital erythropoietic porphyria (CEP) and erythropoietic protoporphyria (EPP and XLPP). These disorders are characterized by deposition of porphyrins in bone marrow and erythrocytes. They typically present early in life with cutaneous photosensitivity.

#### 3.7.1. Congenital Erythropoietic Porphyria (CEP)

##### 3.7.1.1. Pathogenesis

CEP or Gűnther’s disease is a rare autosomal recessive disorder that results from mutations in the gene that encodes for uroporphyrinogen III synthase (URO3S). This gene has been localized to chromosome 10. The disease is of variable severity, depending upon the degree of decrease in URO3S activity. The enzyme activity depends on the causative mutations. More than 35 different mutations have been described. The C73 R is the most common mutation. It affects the stability of UROS protein resulting in its premature degradation. Homoallelic mutation of C73 R or P53L results in a severe phenotype causing hydrops fetalis and transfusion dependency. Heteroallelic mutations (V82F, A104V, A66V, *etc.*) with retention of some enzymatic activity result in milder forms of CEP [96,97,98]. 

Decreased enzymatic activity results in the accumulation of uroporphyrin I and coproporphyrin I in bone marrow, erythrocytes, plasma, urine and feces, as well as deposition in the teeth, bones, skin and other tissues [99].

##### 3.7.1.2. Clinical Features (Table 5)

CEP can present *in utero* with non-immune hydrops fetalis caused by severe hemolytic anemia. Infants may present with neonatal jaundice, and subsequent phototherapy will lead to a severe reaction. Photosensitivity will cause rapid development of vesicles and bullae, which then rupture. Over time, chronic facial scarring, secondary infection and bone resorption can produce significant facial and limb disfigurement.

Other manifestations of CEP can include reddish-brown teeth that fluoresce under long UV light (erythrodontia). Accumulation of porphyrins in erythrocytes can result in hemolysis and eventually hepatosplenomegaly. Late-onset disease has been described in adults.

In the late onset form, clinical features are usually milder, and some adult patients have had an associated myeloid malignancy with normal erythrocyte URO3S activity. It is thought that the variable clinical presentation is dependent upon the degree of enzymatic deficiency.

##### 3.7.1.3. Diagnosis

CEP patients have significantly elevated urinary uroporphyrin I and coproporphyrin I, (100–1000-times normal) and circulating porphyrins (Table 6). Urinary ALA and PBG, meanwhile, are normal. The diagnosis of CEP is made by the identification of specific URO3S gene mutations or deficient URO3S activity. *In utero*, amniotic fluid porphyrins can be measured and URO3S activity can be measured in cultured amniotic cells or chorionic villi [100].

##### 3.7.1.4. Management

The treatment of CEP starts with Sun protective measures. Care should be taken to avoid skin trauma, and early treatment of skin infections is warranted. Chronic erythrocyte transfusions treat anemia and effectively suppress erythropoiesis. However, they can result in iron overload, which may require parenteral deferoxamine or orally-active iron chelators, deferiprone or deferasirox. More invasive measures, such as splenectomy, have been tried to decrease hemolysis and transfusion requirements, but the long-term benefit is still questionable. Bone marrow transplantation has been used to treat several transfusion-dependent children [101,102]. Successful bone marrow replacement is curative [103].

Genetic counseling should be offered to affected families, as URO3S mutations leading to severe phenotypes can be detected prior to birth.

#### 3.7.2. Erythropoietic Protoporphyria (EPP, XLPP)

EPP is an inherited disorder characterized by the accumulation of protoporphyrin in blood, erythrocytes and tissues, leading to painful photosensitivity. It has been reported worldwide with a prevalence of 1:75,000 to 1:200,000 [62]. 

EPP has the distinction of being the most common erythropoietic porphyria, the most common porphyria in children and the second most common cutaneous porphyria after porphyria cutanea tarda.

#### 3.7.2.1. Pathogenesis

Classical EPP usually occurs as a result of the inheritance of a mutated ferrochelatase (*FECH*) allele from one parent and inheritance of a low expression allele (IVS3-48T/C) from the other. The *Fech* gene is located on chromosome 18, and more than 135 *Fech* loss-of-function mutations have been identified. More recently, 5%–10% of subjects with a typical EPP phenotype have instead been found to have an X-linked variant of EPP (termed XLPP) resulting from a gain-of-function mutation of the *Alas2* gene, which have thus far been concentrated in exon 11 [104]. Fech is the final enzyme in heme biosynthesis, and it catalyzes the insertion of iron into the protoporphyrin ring to generate heme (Figure 1). Enzyme activity is reduced to 10%–35% of normal in all tissues, though the excess protoporphyrin is mainly produced in bone marrow.

#### 3.7.2.2. Clinical Features

Similar to CEP, EPP/XLPP has variable clinical expression. The first comprehensive description of EPP was published by Magnus *et al.* in 1961 [105]. Most patients present during infancy or early childhood upon Sun exposure. Accumulation of lipid soluble free protoporphyrin in skin and dermal blood vessels and subsequent photoactivation by sunlight results in the characteristic cutaneous manifestations of EPP. Children with the disorder develop pain, redness, swelling and pruritus within minutes of exposure to sunlight. These symptoms can last for hours to several days. Unlike CEP, vesicles and bullae are uncharacteristic, only occurring in 10% of cases. Chronic skin changes, such as lichenification, pseudovesicles and labial grooving, can develop after repeated photosensitivity episodes. These chronic findings are most prominent in a malar distribution on the face and on the knuckles of the hands.

Patients with EPP and XLPP may develop a variety of hepatobiliary complications. Pigment gallstones, composed partially of protoporphyrin, can cause symptomatic cholelithiasis and biliary obstruction in up to 20% of patients. Protoporphyrin can crystallize in hepatocytes and biliary radicles, impair bile flow and lead to pigmentary cirrhosis in 3%–5% of patients. More mild liver disease characterized by elevated aminotransferases is also seen.

#### 3.7.2.3. Diagnosis (Table 5)

Painful photodermatosis without blistering in an infant/child should raise concern for EPP. With EPP, there are markedly increased levels of protoporphyrin in plasma and red cells. Analysis of plasma porphyrin fluorescence emission (following excitation with light in the Soret band (400–410 nm)) shows a characteristic peak at 634 nm. Since protoporphyrin is not excreted in urine, and urinary porphyrin and porphyrin precursor levels are normal. These biochemical tests can then be confirmed by mutational analyses of the *Fech* and *Alas2* genes. 

Once the diagnosis is made, additional testing should include tests of hepatic function and abdominal ultrasonography to evaluate for hepatobiliary disease.

#### 3.7.2.4. Management

Many treatment options have been employed in an effort to reduce protoporphyrin production in bone marrow and enhance its biliary excretion. Much like CEP, Sun protective measures are an integral part of the management of EPP. Conservative treatment with opaque sunscreens (e.g., zinc oxide, titanium dioxide) and barrier clothing is recommended. Oral beta-carotene (Solatene) has been used, although the clinical benefit is limited, and the yellow color of the skin is not liked by some who have tried it. An alpha-melanocyte stimulating hormone analog (afamelanotide, Clinuvel, Inc.) has been tested recently in placebo-controlled, prospective randomized trials with greater evidence of a benefit, especially improvement in the quality-of-life among EPP patients who have received the active drug [106,107,108].

For those with hepatobiliary disease, bile acid binders, such as cholestyramine (a suggested dose of 4–16 g daily), have been used to interrupt the enterohepatic circulation of protoporphyrin. Ursodiol has been tried to promote the biliary excretion of protoporphyrin [109,110].

Iron supplementation may be considered in patients with low ferritin, but the response to treatment has been variable. Iron administration can reduce porphyrin accumulation by converting it into heme, thus enhancing post-translational stability of *Fech* [111]. There have been reports of improvement [112] of the disease with iron therapy. However, unexplained worsening of photosensitivity has also been noted [113,114]. Thus, the role of iron supplementation in EPP requires additional careful study.

Exchange transfusions, hematin infusions and plasmapheresis have been used to remove protoporphyrin as a bridge to liver transplantation. The proportion of patients who develop severe liver decompensation has not yet been well established. A review of all reported cases of EPP (40 in 2002) estimated that the incidence of patients with EPP who develop significant liver disease is 3%. It should be noted that this is not a population-based estimate. Patients with decompensated liver disease and an MELD score of >15 should be considered for liver transplantation. The first liver transplant for EPP-related liver disease was performed in 1980. One important consideration in EPP patients is that operative lights have been reported to cause third degree skin burns and small bowel perforations. Recommended perioperative management includes the use of special filters to block wavelengths that excite protoporphyrin (400–410 nm) [66].

A U.S. series of 20 patients who underwent liver transplant from 1979 to 2004 for EPP has been reported. Early complications, including postoperative neuropathy requiring prolonged ventilation (six patients) and biliary complications (nine patients) were frequent. However, the 1-, 5- and 10-year survival rates of 85%, 69% and 47% were comparable to transplants done for other indications. Recurrent EPP liver disease occurred in 11 of the 17 patients who survived more than two months after transplant. Recurrence happened as early as eight months after transplant, and three patients were given a re-transplantation [115]. Because, in EPP, the bone marrow, rather than the liver, is the chief source of excess protoporphyrin production, successful bone marrow transplantation would be expected to correct the critical metabolic defects. 

Bone marrow transplantation for EPP is generally not considered unless there is evidence of significant hepatic injury, in which case, it is considered in conjunction with liver transplantation [115].

## 4. Conclusions

It is important that physicians consider the possibility of porphyria when they see patients with symptoms that may be compatible. The most common presenting symptoms are recurrent episodes of severe abdominal pain, especially occurring in women aged 18–45 years, or evidence of acute or chronic photosensitivity.

The single most important test to establish or exclude a diagnosis of an acute porphyria is a rapid test for PBG in the urine. This test should be available and should be run every day of the week, with rapid turn-around times at all major centers, because of the importance of ruling-in or ruling-out the diagnosis of acute porphyria.

The most important tests when cutaneous porphyria is suspected are plasma and erythrocyte porphyrin levels. If they are increased, additional studies, including genetic testing, should be performed in order to establish the specific diagnosis.
